# Patterning Fidelity Enhancement and Aberration Mitigation in EUV Lithography Through Source–Mask Optimization

**DOI:** 10.3390/mi16101166

**Published:** 2025-10-14

**Authors:** Qi Wang, Qiang Wu, Ying Li, Xianhe Liu, Yanli Li

**Affiliations:** 1 School of Micro-Electronics, Fudan University, Shanghai 200433, China; wangqi_fd@fudan.edu.cn (Q.W.); wu_qiang@fudan.edu.cn (Q.W.); 24112020146@m.fudan.edu.cn (Y.L.); xianheliu@fudan.edu.cn (X.L.); 2 National Integrated Circuit Innovation Center, Shanghai 201204, China

**Keywords:** Source–Mask Optimization, computational lithography, lithography imaging model, pattern fidelity, aberration control

## Abstract

Extreme ultraviolet (EUV) lithography faces critical challenges in aberration control and patterning fidelity as technology nodes shrink below 3 nm. This work demonstrates how Source–Mask Optimization (SMO) simultaneously addresses both illumination and mask design to enhance pattern transfer accuracy and mitigate aberrations. Through a comprehensive optimization framework incorporating key process metrics, including critical dimension (CD), exposure latitude (EL), and mask error factor (MEF), we achieve significant improvements in imaging quality and process window for 40 nm minimum pitch patterns, representative of 2 nm node back-end-of-line (BEOL) requirements. Our analysis reveals that intelligent SMO implementation not only enables robust patterning solutions but also compensates for inherent EUV aberrations by balancing source characteristics with mask modifications. On average, our results show a 4.02% reduction in CD uniformity variation, concurrent with a 1.48% improvement in exposure latitude and a 5.45% reduction in MEF. The proposed methodology provides actionable insights for aberration-aware SMO strategies, offering a pathway to maintain lithographic performance as feature sizes continue to scale. These results underscore SMO’s indispensable role in advancing EUV lithography capabilities for next-generation semiconductor manufacturing.

## 1. Introduction

Optical lithography serves as a fundamental manufacturing process for producing very large-scale integrated circuits. As the semiconductor industry continues to push the boundaries of miniaturization and performance, extreme ultraviolet lithography (EUVL) has become indispensable for fabricating advanced integrated circuits (ICs) with sub-seven-nanometer node features [1]. The progression toward nanoscale fabrication presents escalating challenges in maintaining both pattern fidelity and process robustness [2]. Modern lithography systems must contend with numerous interdependent parameters that frequently exhibit non-ideal behavior, resulting in complex coupling effects that amplify process variability. A fundamental constraint of lithography originates from the diffraction limit, particularly when implementing aggressive design rules that demand minimum pitches below 40 nm [3].

To address these challenges, Source–Mask Optimization (SMO) has established itself as a critical computational lithography technique since the 22 nm technology node [4]. By simultaneously optimizing both illumination source profiles and mask patterns, SMO enhances resolution while expanding the process window [5,6,7]. Through advanced iterative algorithms that generate optimized freeform illumination sources, SMO effectively mitigates diffraction-induced limitations, enabling robust patterning at reduced pitches while preserving sufficient process window margins. As an extension of EUV lithography capabilities, SMO has demonstrated measurable improvements in image contrast, edge placement accuracy, and overall pattern fidelity, particularly critical for aggressive sub-7 nm node design rules [8,9]. Multiple optimization algorithms [10], including gradient-based methods [11], genetic algorithms [12], and augmented Lagrangian methods [13] have been introduced to significantly accelerate computational speed while maintaining imaging quality [14]. Wu et al. introduce the weighted Zernike polynomials as basis functions to represent the source patterns, suggesting a structural parallel between source optimization and optical aberration characterization [15].

One of the critical challenges in EUV lithography is its aberration controls [16]. Aberration refers to the deviation in an actual wavefront from its ideal shape, resulting in significant discrepancies between observed imaging results and theoretical expectations. Optical aberrations in the lithography system will introduce wavefront distortions that degrade pattern fidelity. The wavefront aberrations are conventionally decomposed into orthogonal Zernike polynomials within circular pupil coordinates. This orthogonal decomposition provides an efficient mathematical framework for quantifying the distinct impacts of various aberration components on imaging performance [17]. For effective aberration control, scanners must minimize the coefficients of individual Zernike to realize aberration compensation and optimization. Therefore, adaptive optics with adjustable mirrors and stages has been employed to dynamically correct lower-order aberrations, while advanced wavefront sensors and optimization strategies are used to further mitigate higher-order components [18]. The compensation approach enables sub-nanometer wavefront accuracy, though practical limitations remain in simultaneously optimizing all aberration modes across the entire exposure field [19]. Nevertheless, current technological limitations prevent complete aberration elimination, resulting in residual aberrations that inevitably impact lithographic performance.

In the following sections, we demonstrate SMO’s capability to compensate for aberration-induced imaging degradation. First, we model forward aerial image formation in partially coherent systems using Abbe imaging theory with Zernike coefficient analysis. Then the SMO framework and its implementation methodology are presented. Subsequent analysis quantifies process window enhancement through SMO optimization. The study further characterizes aerial image distortion under typical lens aberration conditions. Finally, we employ SMO to actively compensate for wavefront distortions, achieving both measurable critical dimension error reduction and notable process window improvement. Crucially, our results demonstrate that SMO effectively compensates for both isolated aberration and complex mixed-mode errors incorporating random higher-order components. This performance establishes SMO as an essential complement to hardware-based aberration correction approaches, ultimately enabling the sub-nanometer patterning fidelity required for sub-7 nm node manufacturing.

## 2. Theory and Methodology

### 2.1. Hardware Foundation of SMO

**Figure 1:**
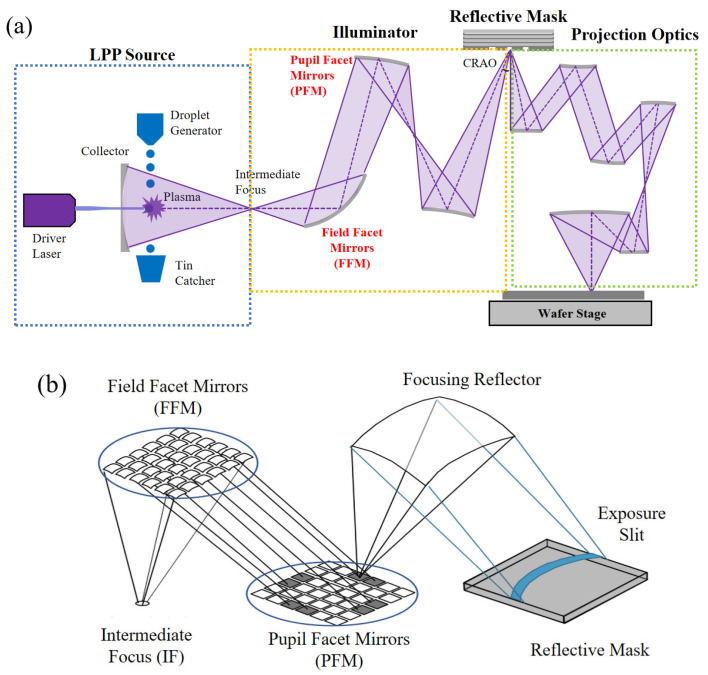
Schematic diagrams of (**a**) EUV lithographic imaging system architecture and (**b**) illumination subsystem components of field facet mirror (FFM) and pupil facet mirror (PFM) configurations.

Figure 1a illustrates the EUV imaging system architecture. In this system, light from the EUV source illuminates the reflective mask through the illumination optics at a specified chief ray angle at object (CRAO, 6° oblique incidence for 0.33 NA EUV). The projection optics then collects and recombines the diffracted orders generated by mask reflection, ultimately transferring the patterned information to the wafer plane. However, due to the diffraction-limited nature of the optical system and the complex physicochemical processes occurring in the resist layer, the printed wafer patterns will exhibit distortions relative to the target design.

The hardware implementation of SMO, illustrated in Figure 1b, is physically implemented through a dual-mirror illumination system consisting of field facet mirrors (FFMs) and pupil facet mirrors (PFMs). Through independent rotation of each facet mirror, the system achieves tailored illumination configurations, with the FFM governing mask-plane uniformity and the PFM dictating pupil-plane source characteristics. The EUV lithography illumination system employs a 49 × 49 array of mutually independent pixels based on the PFM grid structure, enabling precise angular control of EUV radiation for generating optimized source profiles. The synchronized operation of these mirror arrays affords the essential degrees of freedom required to realize illumination customization that is critical for SMO’s wavefront correction capabilities [20,21].

### 2.2. EUV Abbe Imaging Model

Source–Mask Optimization fundamentally relies on computational analysis of the aerial image intensity distribution at the wafer plane for specified source and mask configurations [22]. According to Abbe’s theory of partially coherent imaging, the aerial image intensity *I(x,y)* is given by the following equation:

(1) $$I\left(x,y\right)=\iint_{\infty }S\left(f,g\right)\left[{\left|\iint_{\infty }H\left(f+f^{\prime },g+g^{\prime }\right)O\left(f^{\prime },g^{\prime }\right)e^{-i2\pi \left(f^{\prime }x+g^{\prime }y\right)}df^{\prime }dg^{\prime }\right|}^{2}\right]dfdg,$$

where (x*, y*) denote the normalized spatial coordinates in the image plane, (f, g) represent the normalized spatial frequency coordinates in the pupil plane, and ($f^{\prime }$, $g^{\prime }$) correspond to the normalized spatial frequency coordinates of the diffracted orders. The aerial image intensity *I* is determined by the Fourier transforms of the source pattern *S*, the pupil function *H* of the projection optics (which acts as a low-pass filter), and the mask’s diffraction spectrum *O* generated through reflection [23]. The overall image is the sum of weighted coherent images [24]. As evident, aberrations directly influence the pupil function, whereas SMO compensates by modifying both the pupil and mask functions. This inherent correlation fundamentally enables SMO to effectively mitigate aberrations in lithography systems.

**Figure 2:**
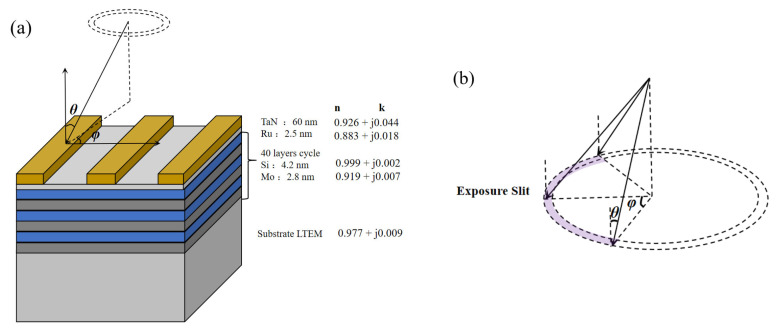
(**a**) The structure of the EUV mask, where *θ* denotes the incident angle of light and *φ* represents the azimuth angle. A typical EUV mask primarily consists of a TaN absorber and a Si/Mo multilayer. (**b**) The schematic of the exposure ring slit in the EUV system. Along the ring slit, the chief ray angle of incidence (CRAO) remains constant at 6°, while the azimuth angle varies from −30° to 30°.

In DUV lithography, the diffraction spectrum *O*($f^{\prime }$, $g^{\prime }$) is conventionally computed using the Kirchhoff thin-mask approximation, obtained through the Fourier transform of the mask pattern *M*$\left(x_{m},y_{m}\right)$, where $\left(x_{m},y_{m}\right)$ denote the normalized spatial coordinates in the object plane (mask plane) [25]. The Kirchhoff thin-mask model becomes inadequate for EUV lithography due to oblique incidence effects and pronounced mask 3D topography [26]. Figure 2a illustrates the EUV mask architecture, where *θ* is the incident angle and φ is the azimuthal angle of illumination. The mask structure primarily consists of a TaN absorber layer on the Mo/Si multilayer reflector. Figure 2b depicts the annular illumination slit configuration in EUV systems, maintaining a 6° chief ray angle at object (CRAO) while the azimuth angle varies continuously between −30° and 30°.

To model mask 3D effects in EUV lithography, rigorous coupled-wave analysis (RCWA) is chosen to model the multilayer mask structure (including absorber, multilayer reflector, and substrate). The multilayer will be discretized into a stack of periodic grating layers. RCWA solves Maxwell’s equations by expanding the electromagnetic fields into spatial harmonics in each layer, accounting for near-field diffraction, edge effects, and phase interactions caused by the mask topography. The reflected field is computed by matching boundary conditions at each interface, capturing 3D effects including shadowing and pattern-dependent intensity asymmetry. By incorporating the computed mask 3D effect into the imaging model, RCWA enables accurate prediction of EUV mask 3D performance, considering critical dimension (CD) errors and contrast loss due to mask 3D effects. For efficient simulation, trade-offs between harmonic truncation orders of diffraction, density of layer discretization, grid of segmentation and computational cost have been optimized [27].

### 2.3. Aberrations with Zernike Polynomials

Optical aberrations critically degrade imaging performance, manifesting as pattern placement errors, contrast reduction, and resolution loss. These wavefront distortions are systematically quantified through decomposition into Zernike polynomials, which form a complete orthogonal basis set for representing aberrations. Each Zernike term corresponds to a specific aberration mode: spherical (Z9), coma (Z7, Z8), astigmatism (Z5, Z6), or trefoil (Z10, Z11) and higher-order aberrations. The optical path difference (OPD) of wavefront data is decomposed into Zernike coefficients, which are a sequence of orthogonal basis functions:

(2) $$OPD(\rho ,\theta )=\sum_{l}a_{l}{\;Z}_{l}\left(\rho ,\theta \right)$$

where *Z_l_* is the *l*th Zernike polynomial and $a_{l}$ is the RMS deviation in the coefficient representing aberration strength.

The application of Zernike polynomials in lithography is particularly critical due to the stringent requirements for CD accuracy, feature resolution and process window control in advanced semiconductor manufacturing [28]. In DUV, scanners allow feedback aberration control by tuning and correction through lens alignment or heating, ensuring optimal imaging fidelity for high-resolution patterning. EUV systems’ operation at 13.5 nm wavelength makes them particularly sensitive to wavefront distortions, where even sub-nanometer-scale aberrations can induce significant phase errors that degrade aerial image quality. Conventional optical correction methods alone are inadequate for these conditions, demanding advanced computational compensation strategies. These include optical proximity correction (OPC) to mitigate critical dimension (CD) bias, and SMO to compensate optical aberrations and maintain patterning fidelity [29].

### 2.4. EUV-SMO Build-Up

In SMO, both the illumination source and photomask must be computationally encoded [30]. Conventional source patterns require few parameters for description, resulting in small-scale optimization problems, while freeform sources employ thousands of variables to achieve greater flexibility. EUV source is constructed by discretizing the illumination into a 49 × 49 matrix, where each grayscale pixel corresponds to the intensity of a source point within the continuum range from 0 to 1 [31].

The mask is constructed by parameterizing the mask layout into a pixelated transmission matrix *M(x,y)*, where each element represents the transmission coefficient of the corresponding mask region. For computational efficiency, the mask is typically constrained to binary or discrete transmission levels (e.g., 0/1 for absorbers in EUV masks). To maintain pattern fidelity while reducing optimization variables, the mask model must incorporate 3D effects through RCWA to accurately simulate diffraction behavior under realistic EUV multilayer conditions.

The goal of SMO is to determine the optimal source distribution S(f,g) and mask patterns *M(x,y)* that minimize the cost function *C*, which typically incorporates pattern fidelity, process window robustness, and manufacturability metrics. The difference between the resist image and the target pattern is a key metric for evaluating image fidelity in lithography. The resist image *I*_r_*(x,y)* is derived from the aerial image *I(x,y)* using a sigmoid function that models the resist’s response to light exposure:

(3) $$I_{r}\left(x,y\right)=sig\;\left\{I\left(x,y\right)\right\}\;=\frac{1}{\;1+e^{-\alpha \left[I\left(x,y\right)-t_{r}\right]}},$$

Here, *t_r_* represents the threshold in the photoresist effect, and *α* denotes the steepness of the sigmoid function. The image fidelity term *R_img_* is then defined as follows:

(4) $$R_{img}\left\{S\left(f,g\right)+M(x,y)\right\}=\sum_{x,\;y}{\left\|I_{r}\left(x,y\right)-I_{t}\left(x,y\right)\right\|}_{2}^{2}\;,$$

Source–Mask Optimization process must not only preserve image fidelity but also ensure a sufficient process window for the exposed patterns, including critical metrics such as exposure latitude (EL) and mask error factor (MEF). Only by satisfying these conditions can the imaging quality be guaranteed. Therefore the overall optimization can be formulated as follows:

(5) $$C\;\left\{S\left(f,g\right)+M(x,y)\right\}=arg\;min\;\left\{R_{img}+\tau R_{pw}\right\}\;\;,$$

where τ is a weight assigned to the process window term $R_{pw}$. To achieve this objective, optimization algorithms such as the conjugate gradient method can be iteratively employed to optimize the mask pattern and source shape.

### 2.5. EUV-SMO Flow

**Figure 3:**
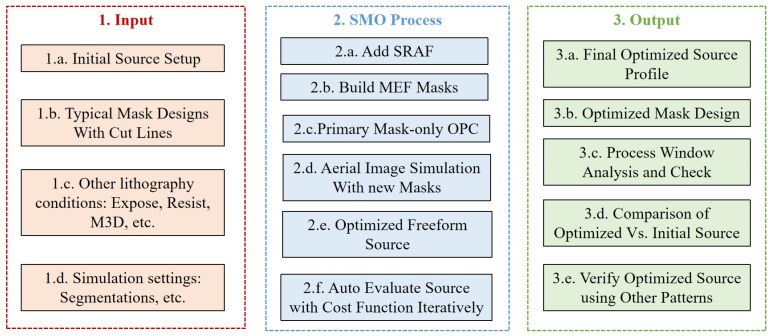
Workflow of the EUV Source–Mask Optimization (SMO).

To investigate the optimization of EUV lithography process metrics and aberrations, we first establish a comprehensive simulation framework, as shown in Figure 3. This framework details the simulation setup, incorporating the SMO input, workflow, and evaluation methodologies to systematically assess SMO’s impact on EUV patterning performance.

The EUV-SMO process begins by defining the representative target layout and setting up an initial source configuration, typically using conventional illumination modes (1.a). The test patterns with cut lines are designed to evaluate imaging quality (1.b), while critical lithography conditions such as exposure dose, resist model, and mask 3D effects (including EUV-specific shadowing and multilayer reflections, 1.c) are accounted for. Other simulation parameters are carefully configured with appropriate segmentation (e.g., 2.5 nm) to balance accuracy and computational efficiency during iterative optimization (1.d).

The SMO procedure concurrently optimizes illumination source profiles and mask pattern parameters. Sub-resolution assist features (SRAFs) are incorporated into the mask design (2.a), while mask error factor (MEF) constraints guide mask modifications to maintain pattern fidelity under defocus conditions (2.b). Primary optical proximity correction (OPC) is first applied to the mask design prior to conducting aerial image simulations with the modified mask patterns (2.c, 2.d). Then, the illumination source progressively develops into an optimized freeform profile through automated evaluation of cost functions that quantify both edge placement errors and image contrast metrics (2.e, 2.f). The optimization framework generates a production-viable source pupil design (3.a) and associated mask corrections (3.b), which are subsequently evaluated through comprehensive process window analysis (3.c) to validate performance stability across diverse test patterns under focus-exposure variations (3.d, 3.e).

## 3. Results and Discussion

### 3.1. SMO Simulation Settings

In SMO, the initial source design constitutes a critical prior that fundamentally governs both the convergence behavior and computational efficiency of the optimization process. Comparing with other conventional source geometries, such as circular, annular, dipole, and quadrupole configurations, quasar illumination has unique advantages for bidirectional pattern design, with iterative adaptive refinement serving to further expand the available process window. Other EUV parameters were configured as follows: numerical aperture (NA) of 0.33, unpolarized illumination, a wavelength of 13.5 nm, exposure energy of 55 mJ/cm^2^, resist thickness of 50 nm (refractive index: 1.0 + 0.025i), photo-acid diffusion length of 4 nm, and DC flare of 5%.

**Figure 4:**
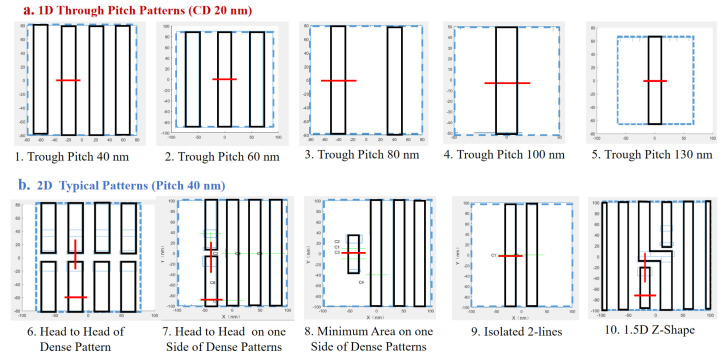
Typical chosen patterns for SMO with cutlines (in red) for 40 nm minimum pitch design rules at 2 nm technology nodes: (**a**) 1D through pitch patterns, (**b**) 2D typical patterns.

We employ representative test patterns to effectively capture the essential challenges of back-end-of-line (BEOL) metal layers while maintaining computational tractability by avoiding full-chip-scale simulations. The selected patterns encapsulate critical 1D and 2D layout configurations to ensure broad optimization applicability. Figure 4 illustrates these patterns with cutlines in red, highlighting dense and isolated features to reflect realistic EUV exposure conditions.

### 3.2. SMO Results

Our Source–Mask Optimization aims to achieve tighter critical dimension (CD) accuracy, enhanced resolution, and an expanded process window. The optimization employs an iterative approach, initialized using empirical estimates of optimal source shapes and mask patterns. The SMO-optimized source is represented as a grayscale pixelated illumination pattern.

**Figure 5:**
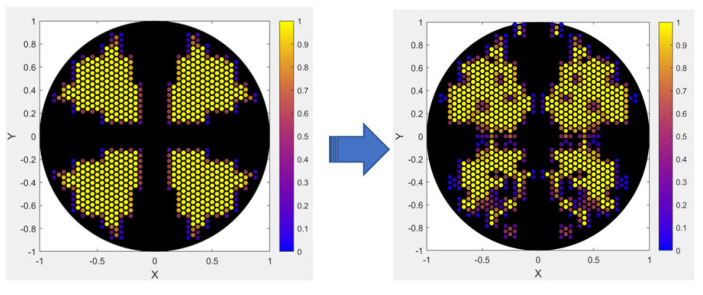
Optimized EUV illumination source pattern derived from quasar configuration, showing grayscale intensity distribution.

**Figure 6:**
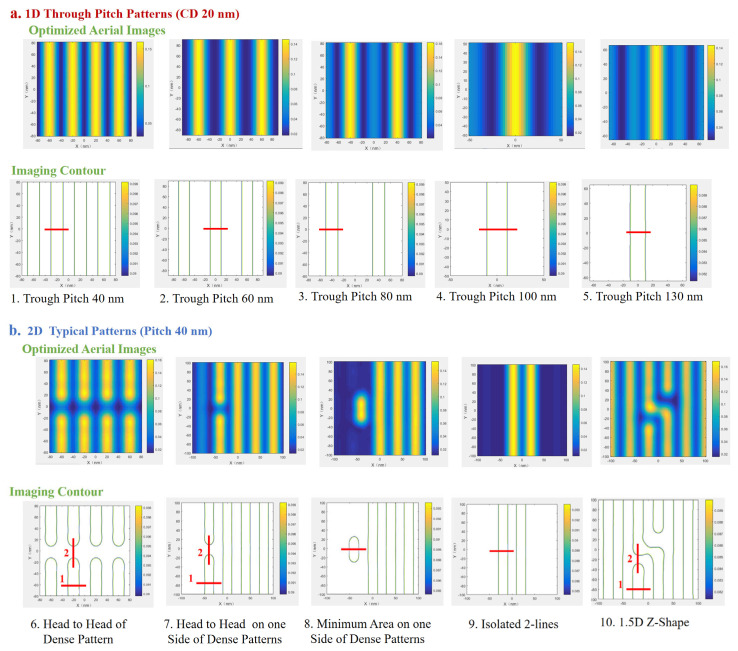
(**a**) 1D and (**b**) 2D aerial images and their corresponding contours, generated using the optimized source and modified masks, Cutlines are shown in red, with numbers indicating the order.

Under the optimized source (Figure 5) and modified mask conditions, the resulting aerial images and their corresponding contours are shown below in Figure 6.

To quantitatively evaluate the SMO-optimized process performance, we employed three critical lithographic metrics: critical dimension error bias, exposure latitude (EL), and mask error factor (MEF). These parameters have been systematically integrated into a multi-objective cost function to drive the optimization toward optimal patterning fidelity.

**Figure 7:**
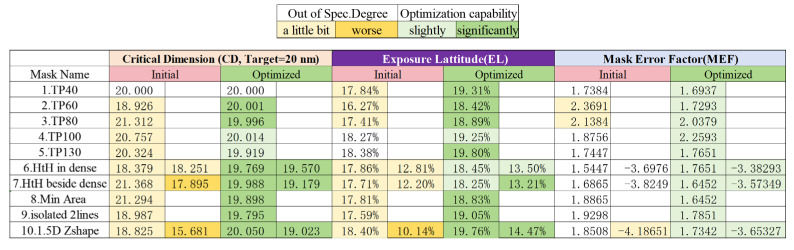
A comparison of critical process metrics before and after SMO.

The critical dimension (CD) variation tolerance is constrained to ≤±5% to ensure pattern fidelity in feature contours. Exposure latitude (EL) specifications are set ≥18% for 1D features and ≥13% for 2D features, representing the permissible imaging contrast. For mask error factor (MEF), the limits are established at |MEF| < 2 for 1D features and |MEF| < 4 for 2D features, quantifying the process sensitivity to mask errors. As shown in Figure 7, pre-optimization results indicate that over half of both CD and EL values exceeded specified tolerances, particularly for complex 2D patterns such as the 1.5D Z-shape. Following three iterative cycles of SMO, all measured parameters conform to the target specifications. This demonstrates that SMO’s efficacy in enhancing lithographic performance, achieving robust pattern reproduction across process variations while maintaining the necessary balance between precision and manufacturability for EUV production applications.

### 3.3. SMO-Driven Aberration Mitigation

Then our study examines the impact of aberrations on EUV imaging performance. By using 1D through-pitch patterns, we analyze how aberrations degrade imaging contrast (affecting exposure latitude, EL). The aberrations include one single order of Z8 = 1 nm RMS and randomly generated aberrations Z_r_ with orders ranging from Z5 to Z26, whose coefficients sum to 1 nm RMS.

**Figure 8:**
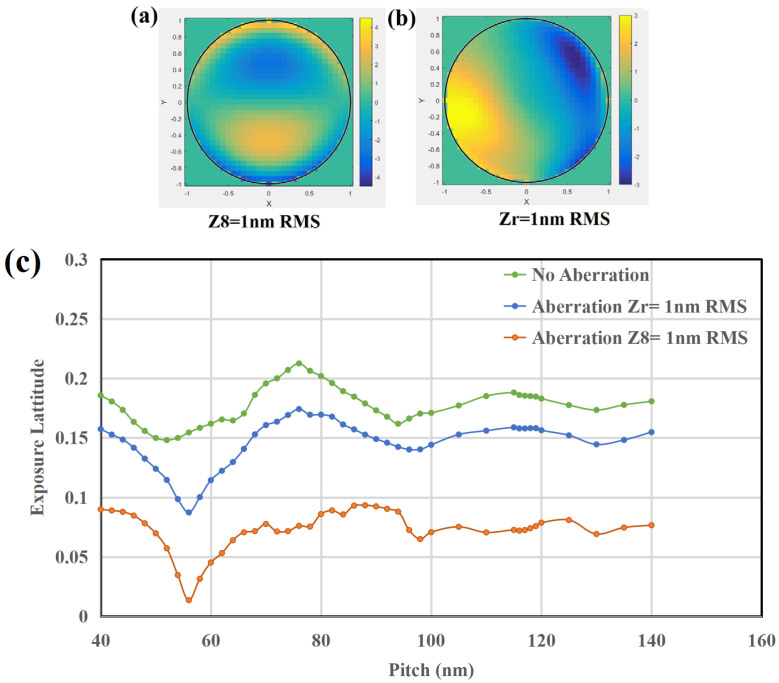
(**a**) A single order of Z8 = 1 nm RMS at the pupil plane, (**b**) randomly generated aberrations Zr with a total of 1 nm RMS at the pupil plane, (**c**) EL variations with 1D through pitch patterns from 40 nm to 140 nm under different aberration conditions.

From the Figure 8 above, it can be observed that the lens’s imaging EL barely meets the 18% production requirement only in the aberration-free case (where the forbidden pitch patterns still fall short). The introduction of aberrations significantly degrades imaging contrast, particularly in the case of a single Z8 aberration.

**Figure 9:**
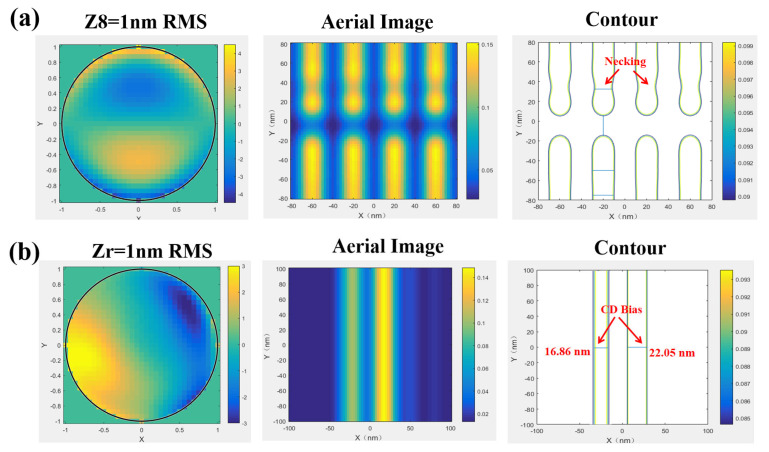
Two cases of 2D typical distortion under aberrations: (**a**) necking in head-to-head patterns and (**b**) CD bias in two isolated lines.

For 2D patterns, we focus on shape distortion effects in critical hot spots, which are particularly sensitive to aberrations. In the Figure 9 below, we present the necking effect on isolated head-to-head patterns under Z8 and CD bias for two-line patterns under Zr. It can be observed that aberrations distort the imaging profiles, thereby degrading the fidelity of 2D patterns.

**Figure 10:**
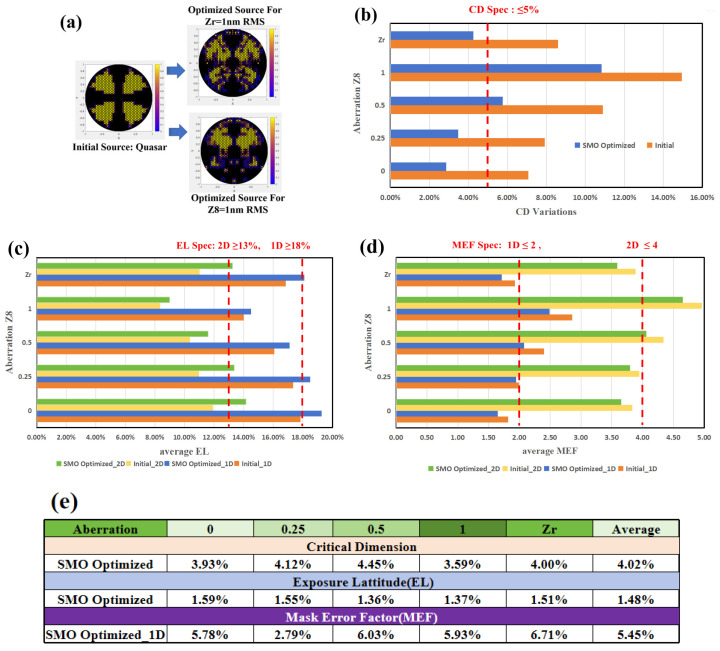
(**a**) SMO from initial quasar source to optimized source for both Z8 and Zr = 1 nm RMS, (**b**) CD accuracy comparison under different aberration conditions before and after SMO, (**c**) EL improvement under different aberration conditions before and after SMO, (**d**) MEF control under different aberration conditions before and after SMO, (**e**) statistical table of CD error reduction, EL improvement and MEF control after SMO.

Next, we examine the impact of applying SMO to aberration-included lithographic process window optimization. SMO optimizations are conducted separately for Z8 (0.25, 0.5, 1 nm RMS) and Zr (1 nm RMS), with the results presented in the Figure 10 below.

The statistical results in Figure 10 demonstrate that SMO improves pattern fidelity and imaging quality under all aberration conditions, yet the optimized process window at Z8 = 1 or 0.5 nm still falls substantially below specifications. In contrast, distributed Zr aberrations totaling 1 nm across multiple orders exhibit significantly less impact on imaging quality. Their effect falls between that of single Z8 = 0.5 nm and 0.25 nm cases. This dispersed aberration situation represents the more practical condition. On average, the CD error was reduced by approximately 4.02%, while EL and MEF have about 1.48% and 5.45% improvement, respectively. Satisfactory production yield is only achieved when Z8 ≤ 0.25 nm. These findings prove that SMO can effectively compensate for image quality degradation when single Z8 aberration remains below 0.25 nm; beyond this threshold, comprehensive scanner-level optimization involving full-system aberration correction and architecture modifications become necessary to achieve required performance.

## 4. Conclusions

This paper proposes a Source–Mask Optimization (SMO) method to enhance extreme ultraviolet lithography patterning fidelity and mitigate aberrations. Through simulations of typical back-end-of-line (BEOL) metal layer patterns, we demonstrate that SMO significantly improves EUVL performance by reducing optical aberration effects, leading to notable gains in critical dimension (CD) accuracy, exposure latitude (EL), and the reduction in mask error factor (MEF). The results indicate that a single Z8 aberration has a more pronounced impact than a randomly dispersed aberration of the same root-mean-square value. Furthermore, Z8 aberrations can only be fully compensated via SMO when their magnitude remains below 0.25 nm. However, this study has certain limitations, including the high computational cost of the SMO process, challenges in scalability to full-chip simulations, and the presence of residual aberrations beyond the compensation range demonstrated here. Future applications of SMO will validate its performance across various IP designs under real-world lithographic conditions, while the integration of Design and Infrastructure, Material, and Process Optimization (DICO) offers a robust strategy to align design intent with manufacturing outcomes, improving patterning fidelity, process window, and yield optimization for advanced nodes. We believe with further refinement of SMO and DICO will be crucial to address the challenges of next-generation semiconductor manufacturing.

## Data Availability

The original contributions presented in this study are included in the article. Further inquiries can be directed to the corresponding author(s).

## Abbreviations

SMO	Source–Mask Optimization
EUV	extreme ultraviolet
IC	integrated circuits
CRAO	chief ray angle at object
FFM	field facet mirrors
PFM	pupil facet mirrors
RCWA	rigorous coupled-wave analysis
CD	critical dimension
NA	numerical aperture
OPC	optical proximity correction
OPD	optical path difference
CDE	Critical Dimension Error
EL	exposure latitude
MEF	mask error factor
